# Hyperdimensional Imaging Contrast Using an Optical Fiber

**DOI:** 10.3390/s21041201

**Published:** 2021-02-09

**Authors:** Jenu V. Chacko, Han Nim Lee, Wenxin Wu, Marisa S. Otegui, Kevin W. Eliceiri

**Affiliations:** 1 Center for Quantitative Cell Imaging, University of Wisconsin, Madison, WI 53706, USA; jenu.chacko@wisc.edu (J.V.C.); hlee768@wisc.edu (H.N.L.); wwu239@wisc.edu (W.W.); otegui@wisc.edu (M.S.O.); 2 Department of Botany, University of Wisconsin, Madison, WI, 53706, USA; 3 Department of Biomedical Engineering, University of Wisconsin, Madison, WI 53706, USA; 4 Department of Medical Physics, University of Wisconsin, Madison, WI 53706, USA; 5 Morgridge Institute for Research, University of Wisconsin, Madison, WI 53706, USA

**Keywords:** hyper dimensional imaging, fluorescence imaging, anthocyanin imaging, hyper dimensional contrast imaging, fluorescence lifetime, polarization, FLIM, anisotropy, hyperspectral imaging

## Abstract

Fluorescence properties of a molecule can be used to study the structural and functional nature of biological processes. Physical properties, including fluorescence lifetime, emission spectrum, emission polarization, and others, help researchers probe a molecule, produce desired effects, and infer causes and consequences. Correlative imaging techniques such as hyperdimensional imaging microscopy (HDIM) combine the physical properties and biochemical states of a fluorophore. Here we present a fiber-based imaging system that can generate hyper-dimensional contrast by combining multiple fluorescence properties into a single fluorescence lifetime decay curve. Fluorescence lifetime imaging microscopy (FLIM) with controlled excitation polarization and temporally dispersed emission can generate a spectrally coded, polarization-filtered lifetime distribution for a pixel. This HDIM scheme generates a better contrast between different molecules than that from individual techniques. This setup uses only a single detector and is simpler to implement, modular, cost-efficient, and adaptable to any existing FLIM microscope. We present higher contrast data from *Arabidopsis thaliana* epidermal cells based on intrinsic anthocyanin emission properties under multiphoton excitation. This work lays the foundation for an alternative hyperdimensional imaging system and demonstrates that contrast-based imaging is useful to study cellular heterogeneity in biological samples.

## 1. Introduction

Hyperdimensional imaging microscopy (HDIM) is a powerful method for combining different fluorescence parameters into discernable combinations of biochemical species [1]. A lack of optimal imaging and computational tools has held back the practical implementations of HDIM, but recent advances in faster computational analyses and imaging techniques have helped advanced this method [2,3]. However, HDIM as an imaging technique still needs speed improvements and better automation to boost performance and adapt to a more lab-friendly version. We have re-engineered the HDIM technique with a sample-specific rationalization targeted towards metabolic and rapid imaging contrast. We study the plant model of *Arabidopsis thaliana* and the intrinsic fluorescence from the stress-induced pigments anthocyanins using HDIM, enhancing the contrast between cells [4]. Anthocyanins are autofluorescent vacuolar pigments with reported antioxidant and photoprotection properties [5,6]. Previous studies of anthocyanin using fluorescence lifetime imaging microscopy (FLIM) have proven effective in understanding anthocyanin trafficking and chemistry [7,8,9]. Our efforts help move HDIM into a real-time tool that is more robust and extends its comparative and contradistinction abilities into disease diagnostics.

There are essential differences between multiparametric approaches and hyperdimensional imaging. Multiparametric approaches often include methods derived from the fundamental parameters, including those that reflect the molecular size, movement, binding state, and others [10,11,12]. A typical example is in the analysis of membrane dynamics. For example, Heikal [13] showed that by combining confocal, FLIM, polarization, and fluorescence correlation spectroscopy (FCS) it is possible to decode membrane thermodynamic derivates such as lipid organization, cholesterol content, protein proximity, mechanical forces, and others. Multiparametric methods can help understand the biological system’s function, learn about its structure, and improve the probing technique as the question evolves over the course of the study. Hyperdimensional methods can read the fundamental fluorescence parameters, including the primary five: intensity, spectrum, polarization, lifetime, and spatial-temporal coordinates [1]. The purpose of HDIM using this direct readout method is to amplify the inherent contrast presented in a biochemical specimen without drawing assumptions on the model of the state or function. Another significant difference is the simultaneous measurement scheme employed in hyperdimensional imaging that can paint ensemble measurements and single-molecule measurements easier to decode. This direct approach gives HDIM an added advantage to pose as a quasi-real-time tool for differentiation in a sample [14]. It is also interesting to note that term Multimodal imaging describes a wide range of non-fluorescence-based techniques or modes of imaging. This list includes not only macroscopic imaging modalities such as magnetic resonance imaging (MRI), optical coherence tomography (OCT), computerized tomography (C.T.), but also the non-imaging modalities such as metabolic profiling and proliferation rates [15].

Fluorescence is a ubiquitous research tool in fields such as biophysics research and clinical diagnostics, spanning a large spatial and temporal ranges of activity. Molecules that emit fluorescence photons unveil their location, orientation, yield, selective underlying energy states, and relaxation times. These five properties are widely used as fluorescence techniques under the common names of fluorescence imaging, fluorescence polarization imaging, fluorescence intensity imaging, fluorescence hyperspectral imaging, and fluorescence lifetime imaging, respectively [12,16,17,18]. A literature review of these methods is outside the scope of this paper, but interested readers are encouraged to pursue these studies [12,19,20,21]. Briefly, fluorescence intensity imaging reveals the spatial and temporal yield of fluorescence photons from a location at a given time. Fluorescence anisotropy or fluorescence polarization imaging identifies the relative polarization state of the emission photons with respect to the excitation [22]. Fluorescence spectral imaging or hyperspectral imaging modalities investigate the fluorescence molecules’ emission spectrum [23]. Fluorescence lifetime imaging calculates the decay rate of a fluorophore [24].

There is only a limited research data available on HDIM contrast, owing to its technical demands. Esposito and Venkitaraman pointed out current limitations in simultaneous observation of physical properties of fluorescence [1]. These authors used grating-based time-resolved spectral detectors on two perpendicularly polarized emission channels. These fast spectral detectors are time-resolved spectrographs made of a multi-cathode array in 16 × 1 or 32 × 1 fashion, coupled to fast-timing electronics units to tag every photon with a time of arrival and the wavelength of origin from each cathode. Using two such detector arrays to tag the polarization states produces a photon with three parameters: time of arrival, a narrow spectral channel of emission, and its polarization state. The individual photon-tags are tallied to make arrival histograms of custom width of resolution. For example, the resulting data for an image of 256 × 256 pixels and 64 time bins is organized in a 16 × 2 × 64 × 256 × 256 matrix (S, P, T, X, Y). S is used to denote Spectrum, P to denote Polarization, T to denote Lifetime, X, and Y for spatial pixel coordinate. This data is analyzed and classified using a dimensionality reduction approach to 3 × 256 × 256 image with the top three components of separation (in the case of principal component analysis (PCA)—based separation). The implementation of HDIM successfully separated the biochemical properties of solutions (rhodamine) and fluorescence imaging samples (convallaria) under varying quenching proportions, viscosity, and polarity [1,25]. Our research attempts to re-engineer the current hyperdimensional imaging set up to accommodate ease of implementation and reduce the burden on computational requirements while revealing biochemical contrast without compromising critical features, such as dynamic range of an image and speed of acquisition. The computational demands from traditional HDIM analysis includes microscopic image matrix compression (data storage), quick histogram calculation (for time-resolved contrast), classification and feature extraction (for spatial selectivity) [26]. Multidimensional matrix calculations are conventionally opted as the solution here; for example, projections of selected axes of interest such as peak-wavelength anisotropy images, and frequency split-hyperspectral images. The choice of dimensionality-reducing methods like PCA, LDA, K-Means, and machine learning techniques such as t-SNE, UMAP, and Autoencoders are often more demanding to implement, let alone extend the method to real-time imaging/visualization [27,28]. Many approaches lighten this load by choosing analysis frameworks such as phasors and other transformation matrices that simplify the multidimensionality into a configurable range of layers of interest [2].

**Figure 1:**
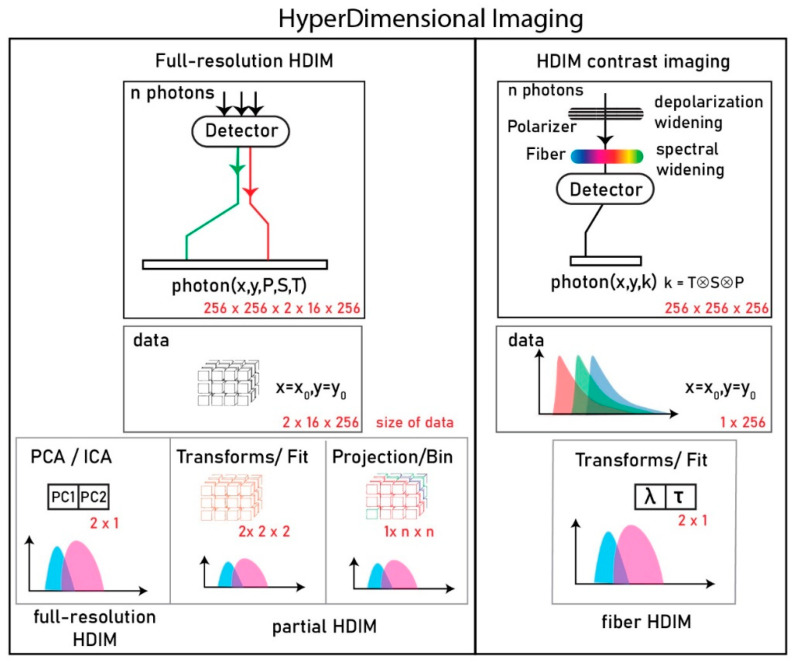
Representation of two different hyperdimensional imaging microscopy (HDIM) collection schemes. The left panel shows the full-resolution HDIM collection that channels multiple photons from multiple detectors through a photon counting module. Each photon is tagged with its position (x,y), polarization (P), Spectrum (S), and Time-lag with respect to excitation pulse (T). An example dataset size is shown in the red font. The data from a single-pixel (x = x0, y = y0) is a 3D distribution (size shown in red font). Three typical routines to analyze this data to achieve a dimension-reduced format shown below. PCA/ICA (principal/independent component analysis), Transforms/Fitting of the curve to derive a limited number of parameters, Projection (e.g., maximum, sum) over an axis or binning over an axis to reduce the size of data. The right panel shows the proposed method where a variable “k” encodes the Time lag (T), Spectrum (S), and Polarization (P) in a single dimension. Compared to full-resolution data, this dataset is just one-dimensional for a single-pixel and can be transformed into a contrast score of interest.

In this work, we show for the first time an optical HDIM setup that derives contrast using an optical fiber-based scheme, resolving underlying species without a substantial computational cost. This system that we named hyper-dimensional contrast imaging is achieved by fixing the axis of time-resolved anisotropy data and convolving the spectral contrast on top of the lifetime signature. The data is collected in 256-time channels for 256 × 256 image size that results in an XYT matrix (256 × 256 × 256), and we paint a contrast based on phasors (2 × X × Y) or custom-gated (1 × X × Y) for real-time analysis. We present a re-engineered version of the previously published HDIM work (full-resolution HDIM) that translates a known fluorescence parameter contrast in a sample of choice into fast imaging [1]. A comparison of full-resolution HDIM and our HDIM-contrast imaging (this paper) is presented in Figure 1.

This manuscript presents our hardware-based dimension reduction method for applying hyperdimensional contrast on *Arabidopsis thaliana* epidermal cells to identify cellular heterogeneity distribution of anthocyanins. Anthocyanins are valuable intrinsic stress-markers, existing in multiple variations and modified forms, including methylated and acetylated forms [29]. Anthocyanins play a photo-protector role against ultraviolet-B radiations in many vegetative tissues and can also act as a free radical scavenger. However, anthocyanin synthesis and sequestration in plant cells are complex and understanding the localization and functional nature of these pigments at the single-cell level has enormous value. The many endogenous fluorophores in plant cells often present challenges in anthocyanin imaging; however, FLIM has shown promise in separating anthocyanins and localizing subcellular pigmentation [8]. We chose this important plant model due to our active work in learning anthocyanin trafficking and function using FLIM [4,7,8,9]. In this research, we present an enhanced plant cell heterogeneity results measured by intrinsic anthocyanins distributions. We demonstrate a fiber-based instrumentation solution for reducing computational complexity associated with hyperdimensional imaging. Our HDIM contrast system can image a biological sample such as *Arabidopsis* tissues with maximized contrast derived from a combination of the fluorophores spectrum, lifetime, and anisotropy.

## 2. Materials and Methods

### 2.1. Optical Setup

We implemented the Hyperdimensional Contrast Imaging scheme using a single time-resolved unit coupled to a fiber-coupled Photo Multiplier Tube (PMT) and a polarization controller using a halfwave plate. We used a previously reported time-resolved FLIM system [30,31] and employed a different emission path for HD-imaging. The time-resolved anisotropy unit remained helpful to verify the ideal polarization states for samples. The microscope light path is custom engineered with a fluorescence lifetime imaging unit (SPC-150, Becker&Hickl Gmbh, Berlin, Germany) and employs an ultrafast laser (MIRA-900, Coherent, Santa Clara, CA, USA) and GaAsP photomultiplier detectors (H7422P-40, Hamamatsu Photonics, Hamamatsu, Japan). The imaging was carried out at 830 nm excitation. Two objective lenses were used (10×, 0.5NA, and 20×, 0.75NA Nikon lenses) for the imaging. The lifetime imaging unit uses a photodiode (PD) to precisely time the laser pulse and measure the time-lag of the arrival of the fluorescence photon from the PMT. The microscope and lifetime acquisition are controlled using a lab-developed software that is capable of laser scanning, image acquisition, and FLIM called OpenScan (unpublished), based on the well-established open source image acquisition software MicroManager [32].

**Figure 2:**
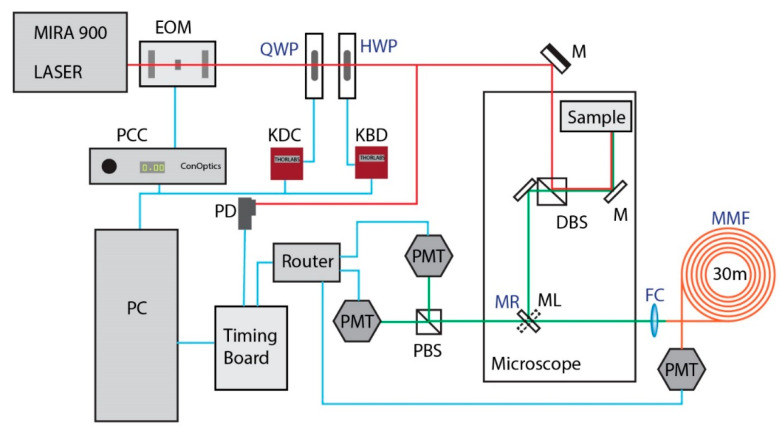
Optical schematic for the fiber-HDIM setup. The laser (Coherent MIRA) passes through an Electro-optic Modulator (EOM) and a polarization control unit that consists of a quarter waveplate (QWP) and a half wave plate (HWP). The light is guided using a mirror (M) into the microscope, and a dichroic beamsplitter is used to split the fluorescence from the excitation light. The emission travels to the port selection mirror right/left (MR-ML). The MR port sends the light to a fiber coupler (FC) and a multimode fiber (MMF) to the Photomultiplier tube (PMT). The ML port directs the light to a polarizing beam splitter (PBS) and two PMTs collecting photons at orthogonal polarization states. The ML port is only used for validation. The PMT signals from all ports and channeled to the router and read by a timing board. The timing board also compares the laser excitation timing from a photodiode (PD) and calculates arrival times of photon. The ML port is an anisotropic validation system, and the MR port is the fiber-HDIM system. The timing information is fed to the PC (computer), which can control the spatial position of the laser and map an image using Micromanager (MM). The EOM is controlled using a Pockels cell Controller (PCC), and the waveplates are motorized using Thorlabs Kinesis motor drivers KDC and KBD based on whether a D.C. or Brushless Motor is used for rotation.

The optical schematic is presented in Figure 2. The spectral contrast is derived using an optical fiber (Fujikura GRIN fiber 800 µm-diameter, 30 m-long, Fujikura, Tokyo, Japan) collection of light, which produces a dispersion of input wavelengths [33]. The dispersion was calibrated for this fiber in the previous publication as 10 nm wavelength for every unit 200 ps shift in time [33]. The spectral-dispersion has been described elsewhere [33]. Briefly, the scanning emission light is focused on the 800 µm diameter fiber using a fiber coupler. The light travels 30 m of glass, where the lifetime decay is convolved with the spectral dispersion. The temporal response thus reflects the decay of the molecule shifted by the spectral peak of emission. This spectrum convolved lifetime decay curve can be analyzed to extract spectral maximum and lifetime separately.

Anisotropy measurements can be either time-resolved or steady-state. For HD contrast imaging, we do not need a complete anisotropy setup. For HDIM, the Anisotropy acquisition is reduced to an axis that provides the best contrast to the spectral detection and can be performed without the extensive two-polarized detector setup. However, we used the anisotropy setup to validate our imaging contrast. In order to study the time-resolved anisotropy parameters, we used the two-PMT setup using a polarizing beam splitter (PBS) on the left port (ML, mirror to left port) shown in the optical schematic (Figure 2). We used a fast brushless motor (KBD101, Thorlabs, Newton, NJ, USA) to rotate a halfwave plate (HWP) and a DC motor (KDC101, Thorlabs, Newton, NJ, USA) to move a quarter waveplate (QWP) to control excitation polarization. We measured a full spectrum of HWP-QWP and estimated the maximum contrast generated in static anisotropy values.

The HDIM imaging optics were installed on the right port of the microscope (Nikon Eclipse TI, Japan) using the MR (mirror to right port) and the excitation polarization control using the QWP-HWP unit. In the case of the *Arabidopsis thaliana* HDIM imaging experiments, a spectral bandpass filter of 630/69 nm (FF01-630/69 Semrock, Rochester, NY, USA) was added to improve the spectral contrast for the anthocyanins. The polarization state was optimized to get maximum contrast between the cells in the image. We used a combination of 160° HWP and 80° QWP angle. The critical parts of our HDIM contrast system are shown in blue font in Figure 2.

### 2.2. Plant Culture

*Arabidopsis thaliana* seeds (Col-0) were sterilized in 10% bleach and 1% Triton X-100 for 1 h and washed six times with autoclaved water. Seedlings were grown in mAIC (modified anthocyanin inductive condition) containing half-strength liquid Murashige and Skoog medium supplemented with 5% [*w*/*v*] sucrose on a rotary shaker with constant light of 200 mol m^2^ s^−1^ at 22 °C [7]. Cotyledons (embryo leaves) were imaged 5 to 7 days after germination.

**Figure 3:**
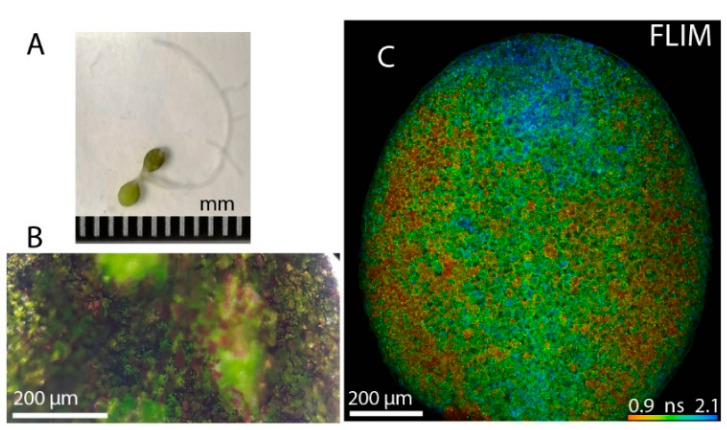
*Arabidopsis* cotyledons and fluorescence lifetime imaging microscopy (FLIM). (**A**) Photograph of an Arabidopsis seedling showing anthocyanin accumulation in its cotyledons. The scale is 1 mm (**B**) A zoomed-in photograph of the cotyledon lower epidermis shows cells with different amounts of anthocyanins (purple shade). (**C**) FLIM image of the cotyledon lower epidermis, where single epidermal cells can be distinguished. The lower lifetimes (red color) correspond to anthocyanins. The scale bar is 200 micrometers, and the FLIM color bar is set for the mean lifetime image from 0.9 ns to 2.1 ns extracted from a multiexponential fit of the data.

*Arabidopsis* cotyledons and anthocyanin distribution patterns are shown in Figure 3. The regions of the cotyledons with anthocyanins are visible in the brightfield as darker/purple areas (see zoomed-in image, Figure 3B). The FLIM image of the lower side of the cotyledon (abaxial epidermis) shows anthocyanin distribution (low lifetime values). A more comprehensive characterization of FLIM of anthocyanin was reported previously [7,8].

### 2.3. Image Analysis

The data for HDIM was collected using a lab-based laser scanning/data acquisition unit based on Micromanager [32]. This module is named OpenScan (unpublished), controls all the motor and galvo functions, and generates time-tagged photons from the timing board electronics (SPC150 and SPCM64 control software, Becker & Hickl, Berlin, Germany) as *.spc and *.sdt files. The files were read in Python and analyzed using FLIMJ [34]. The intensity images were segmented using Cellpose [35] and mapped to the multiparametric images. The colormaps were applied using Fiji [36] look-up-tables, and each leaf was mapped to the mean value presented from the set of pixels in each leaf-cell ROI (region of interest). The unsegmented cells were omitted for all analyses. All the results presented in this manuscript are from a single leaf image, focusing on single-cell separation. The imaging was tested on multiple (n > 6) leaves for reproducibility, but not presented. The PCA, LDA analysis was done using the scikit-learn Python package [37]. The k-means and silhouette scores analysis for phasors were also carried out using the same package and were described previously [38].

## 3. Results

HDIM imaging modalities were briefly discussed in the introduction section (Figure 1). The full-resolution method of collection, as described by Esposito, allows a complete analysis of the photons. The photons analyzed paint a pixel in discriminating fractions of different species, plotting kinetic changes over time, or similar biochemical changes. This contrast in a pixel results from discrimination drawn between photons along n-number of axes (for an n-dim PCA reduction). In some instances where principal axis #1 (PC1) alone brings the amount of variance of the transform to 99% of the original dimensions, the contrast lies mainly in one axis drawn across an n-dim tensor space of spectrum-anisotropy-lifetime space. This axis of separation can be partially simulated using hardware by controlled anisotropy-spectral collection. Otherwise, there exists a set of polarization, spectral, lifetime states that generate a maximum difference between species in a pixel. When extended in an image, these static values help the pixel distribution generate maximum contrast between pixels.

For fiber-HDIM contrast imaging, we find the best emission polarization contrast and fix the polarization state to optimize the anisotropy-spectral contrast. We do this by tuning the excitation polarization instead of emission because the dichroic on emission paths has its polarization preference. Moreover, controlling excitation polarization gives better control of the system. The emission photon’s spectral position is encoded as a delay using fiber optics [33]. The spectral resolution can be varied by changing the fiber length, and we found 30 m length suitable for our experiments. The convolution of photon’s spectral information on the lifetime-decay curve makes the dynamic information content in the decay curve higher. This decay curve is fit using an exponential-curve fit or transformed using phasors to paint contrast in the image. However, the spectral convolution is selectively biased by the dominating fraction in the pixel. The exponential intensity decay curve can separate multiple species by virtue of fitting; however, the spectral information coded on it is challenging to be separated.

The signal in the lifetime curve is a polarization filtered spectrally convolved intensity decay curve in the order of nanoseconds. The two values that need to be set for a particular unknown sample is the best anisotropy contrast (aka the excitation polarization angle) and appropriate spectral bandpass filter (to restrict the spectral broadening to a smaller range). Once obtained for a sample, these optimal values of polarization and spectral range can be used as a priori information for fiber-HDIM to operate at a full-imaging pace. This step of identification of the tensor axis is derivable even by the sequential approach to formulating the P-S-T matrix and fix the best P-S values that aid the image contrast. In the case of *Arabidopsis* leaves, we did a sequential collection and set the spectral filter at 630/69 nm and polarization angle combination of HWP: QWP at (160:80°). The polarization optimization was carried out the entire 360°–360° degree matrix for static anisotropy contrast because there was variability in the peak contrast obtained each unit of (90:45°) combination. This variation is possibly due to imperfect collection and delivery of polarized light.

The following results show a PCA analysis of a sequential collection of time-lag, anisotropy, and spectral-peak using the fiber. We also show how a spectral bandpass filter helps in improving the contrast. Finally, when used with the bandpass spectral filter, the direct analysis of the “polarization filtered spectrally convolved intensity decay curve” from the fiber yields a better contrast than the sequential collection PCA analysis.

### 3.1. Hyperdimensional Imaging Using Derived Parameters

**Figure 4:**
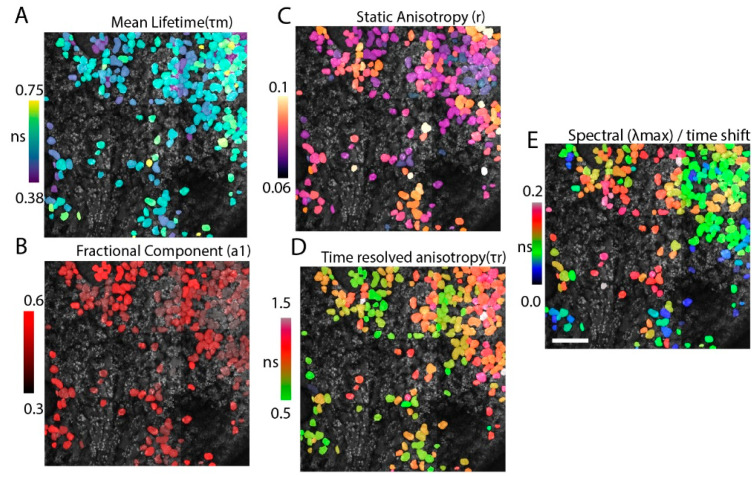
Multiparametric Collection. The contrast from different fluorescence modalities is compared here. The images are segmented for cotyledon epidermal cells, and each parameter per-cell is colored and overlaid on the intensity image (in grayscale). (**A**) the lifetime curves are fit using a 5 × 5 kernel size and fit to multiexponential fits. The mean lifetime is colored in this scheme. (**B**) The fractional component of the smallest lifetime species (anthocyanins) is shown. (**C**) The static anisotropy parameter (r) derived as a ratio of depolarized light to total emission light is shown here. (**D**) The time-resolved anisotropy curve (r(t)) is fit to multiexponential fit and plotted for the mean rotational time. Note that these values are higher than anthocyanin lifetime, and only the smallest (green) values represent anthocyanins. (**E**) The peak-wavelength parameter is derived from the fiber-shift calculation and overlaid as previous panels. Panels C and B are ratio-metric quantities without units. All five panels show different parameters, showing different contrast between cells.

In order to validate the HDIM contrast, we measured the different parameters sequentially. We selected a smaller areas of the cotyledon lower epidermis and (a) calculated the mean lifetime using a bi-exponential decay; (b) the fractional distribution of lifetime species; (c) measured the static anisotropy using two orthogonally polarized detectors and calculating the intensity ratio; (d) calculated the rotational time using anisotropy decay fit into an associated anisotropy decay model using two lifetime values; and finally, (e) imaged using the fiber to measure spectral peaks (encoded as time-shifts). The five resultant images were analyzed using a segmented intensity map to paint single leaf-cells with the respective parameter’s mean value. The images are presented in Figure 4.

**Figure 5:**
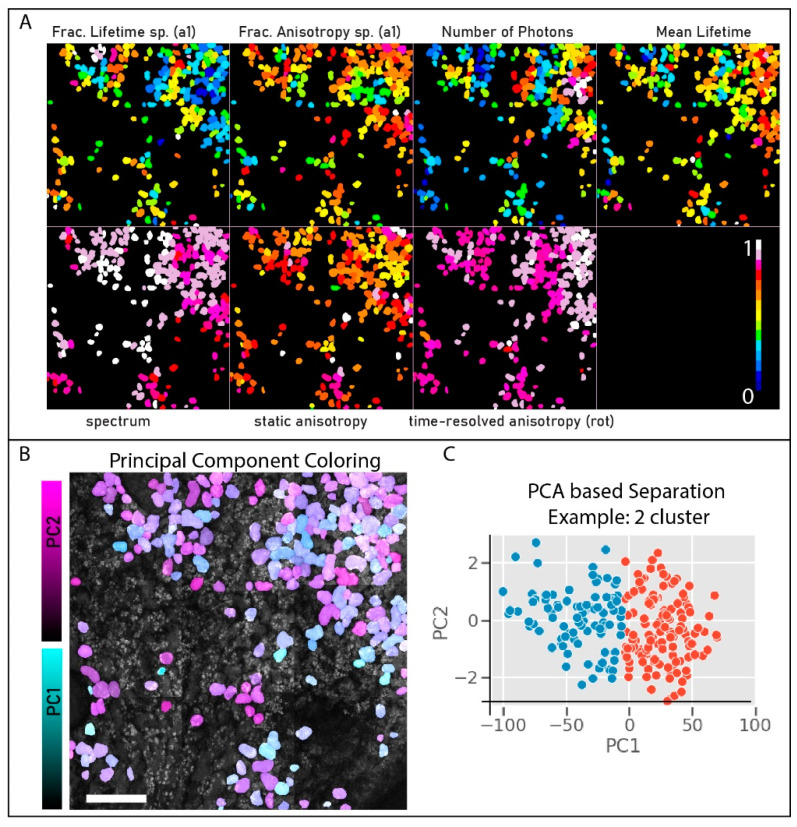
HDIM Computational Approach. The HDIM computation includes processing of large lifetime-histograms mapped to spectrum, and polarization read as a multidimensional data set (16 ch × 2 pol × 256) using a principal component analysis. (**A**) The seven derived inputs for PCA analysis are shown here. This includes fractional lifetime of the lowest lifetime species (a1), fractional rotational anisotropy of the lowest lifetime species(a1), intensity, mean fluorescence lifetime, spectral-peak wavelengths, static anisotropy values and time-resolved mean rotational time. This analysis reduces the data into three components, often painted in RGB. In this dataset, the derived parameters are fed into the PCA analysis, and only two components are derived, PC1, and PC2. The two components are painted in cyan and magenta and overlaid on the intensity image in panel (**B**). The two components were analyzed by multiple cluster separation using a k-means separation. A 2-component separation is shown in panel (**C**) as an example.

We used the five parameters shown in Figure 4, along with the intensity data and the fractional anisotropy data, into a sparse-PCA. This reduced them into two components: principal component #1, #2: PC1, PC2) [39]. Although we could perform PCA by using the 2 (P) × 256 (T) × 256 (S) dataset from a single pixel, the comparison would not be fair because the fiber-based spectral collection is not a full-hyperspectral imaging unit. The [S] dimension has the lifetime and spectral information are convolved on each other. The seven parameters were standardized (as shown in Figure 5A), and then two axes of separation were derived and colored per leaf-cell (Figure 5B). The PCA scatter points were further analyzed using a variable k-means clustering algorithm to identify clusters, and no satisfying separation of clusters (measured using silhouette score) was obtained. The two-component k-means separation is shown in Figure 5C.

This method was a means to understand if we could derive a satisfying PCA axis using sequential measurement. This analysis is not perfect; however, standardizing this way helps us deduce which variables offer the most contrast. Although we present only seven parameters, a total of 25 derived parameters were tested as PCA dimensions. This list includes the fit parameters from both time-resolved anisotropy fit and lifetime fit, their fit-goodness values (by a chi-square value), intensity offset, mean-lifetime (t_m_), and intensity weighted-mean lifetime (it_m_). The 25 variables were (T: (t_1_, t_2_, a_1_, a_2_, f_1_%, offset, chi-sq, it_m_, t_m_, sum), P: (t_1_, t_2_, a_1_, a_2_, f_1_%, offset, chi-sq, it_m_, t_m_), P (r-static, r_0_, r-infinity), S (peak-shift, peak-width, offset)). These additional parameters did not help the PCA separation; instead affected the noise covariance and lowered the cumulative explained variance ratio of all components [37].

### 3.2. Single-Shot HDIM

**Figure 6:**
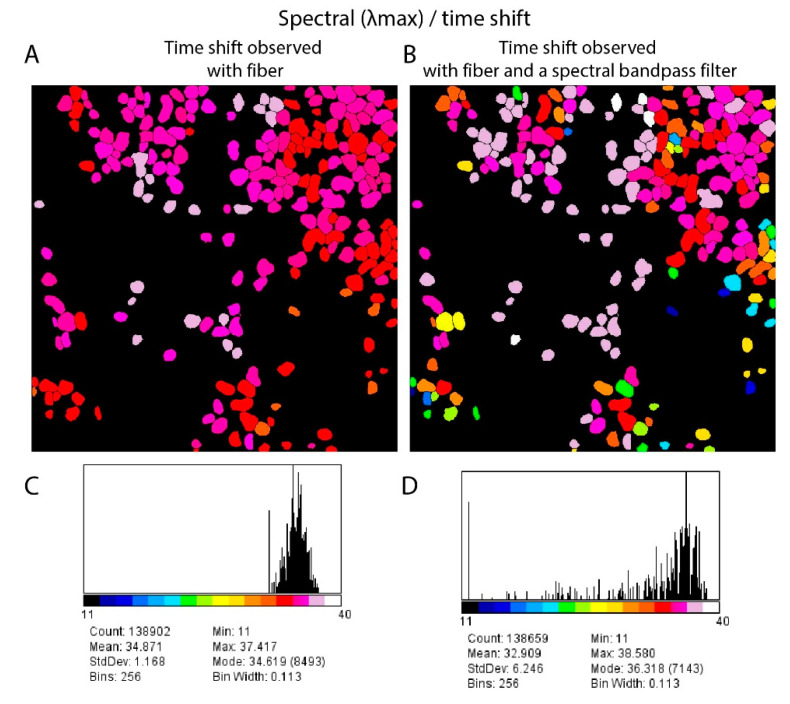
Spectral Contrast using a filter. The spectral peak mapping using fiber dispersion and timing electronics is shown in panel (**A**). The same area gets a larger contrast when a 630/69 nm filter is added before the fiber (panel **B**). Comparing both image histograms is shown in panels (**C**) and (**D**) for panels (**A**) and (**B**), respectively. The images are 1.14 mm in size and use the same segmentation scheme shown before. The color scales of panels (**A**) and (**B**) are shown along the histograms presented in panels (**C**) and (**D**).

The spectral information derived using the fiber was further studied for increasing the HDIM contrast. The highest photon emitter often dominates the spectral widening of the lifetime curve in a single pixel. This limits the separation to a small range because most cells default to the anthocyanin spectral peak. We found that the spectral distribution widens when we collect in a smaller spectral range by adding an emission filter. This spectral-band restriction of total fluorescence photons allows more contrast between pixels owing to minor changes in spectral peaks. A comparison of time-shift due to dispersion with and without a spectral bandpass filter is shown in Figure 6. The time-shift images of leaf-cells in Figure 6 (A: without filter, B: with filter) is a smaller distribution, while with addition of the 630/69 nm filter, the spectral peaks time-shifts) are broadly distributed to a six-time wider distribution (Figure 6C,D). Although this causes a reduction in total available photons, the enhancement of cellular contrast is significant.

### 3.3. Comparing Fiber HDIM vs. Derived Parameters HDIM

**Figure 7:**
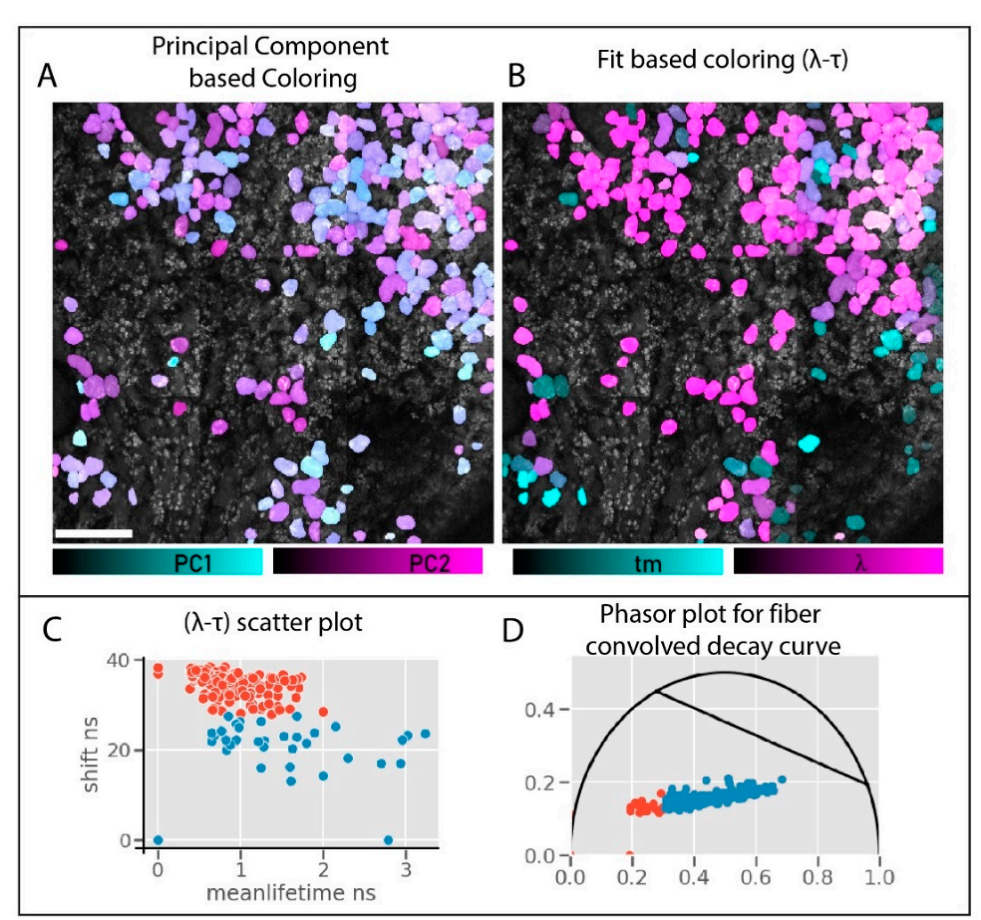
HDIM PCA vs. fiber-HDIM. The PCA analysis result (**A**) is compared against the mean lifetime -spectral shift map (lambda-tau) map (**B**). The PC1-PC2 colors separate the plant cells poorly; however, lambda-tau maps show separation and further distinguish anthocyanin distribution better (panel **B**). The 2D scatter plot of the cells in panel (**B**) is shown in panel (**C**). The two separate clusters are visualized. Panel (**D**) shows a fit-free analysis of the spectrally coded lifetime data, which resolves two species. These cells (blue-scatter plot in phasor) are prominent in magenta color in panel (**B**), show a relatively lower lifetime, and represent a higher anthocyanin accumulation. The black line on the phasor plot is shown as a reference for 0.5 ns and 3.0 ns as the regular anthocyanin lifetime limits.

This spectral selection and polarization optimization help us reduce the collection to a single channel. The single detector connected to the fiber is used for fiber-HDIM. We compare the results from the fiber-HDIM with the derived-parameter PCA output in Figure 7. The intensity decay curve with maximum anisotropy contrast and selected spectral range is analyzed in two different schemes. (1) The time-shift is derived as lambda max and plotted against the fitted mean-lifetime value: this 2D histogram plot is called lambda-tau map (following the naming from previous work) [33]. (2) The data can be transformed into a phasor plot where both shift and lifetime will significantly affect the transform. These two schemes are shown in Figure 7C,D. The λ-τ (lambda-tau) values can be color-coded on the leaf cells for maximum contrast. (This can also be done using g-s of phasor transform, not shown). We compare (Figure 7A,B) the λ-τ (lambda-tau) map contrast against the derived PCA contrast obtained before (Figure 5). We can identify two distribution and easily separable HDIM contrast using the λ-τ maps. Moreover, this fitting process is fast (in milliseconds [34]) and does not rely on extensive data processing, making this a real-time contrast modality in imaging.

The enhanced contrast can be compared by comparing the scatter plot from PCA analysis Figure 5C and λ-τ maps in Figure 7C. The PCA separation is hardly noticeable, while the scatter plots show a more apparent separation. The phasor plot in Figure 7D shows a separation with a good silhouette score (>0.5).

## 4. Discussion

Fluorescence-based studies harness the physical properties of fluorescence that change with microenvironmental changes of the molecule. Different properties enable us to study a chemical event of interest with different perceptions. Anisotropic studies often lead to an understanding of orientation and extend into the rotation, size, and binding state of that molecule. Similarly, spectral and decay rate studies unveil chemical events in relation to temperature, viscosity, and molecular conformation. Most biophysical studies limit their work to one of these fluorescence properties, which often produce contrast in their images and study the problem. In order to learn more details about the molecule, all the molecular properties need to be mapped out and analyzed in a hyper-dimensional space—furthermore, either a dimension reduction method or a multi-dimensional analysis to extract parameters of interest must be employed. These methods, although not difficult to deploy, are often time-consuming and expensive. Here we give an alternate option where a hyperdimensional contrast from multiple fluorescence properties can be encoded into a single observation using a single detector.

Fluorescence can reveal molecular binding, molecular selectivity, and physical behaviors such as rotation, translational restrictions, channeling, and environmental changes such as temperature and viscosity. Most biological system studies often confound in this multidimensional information space because extensive studies require a longer time and additional research to validate the properties. We propose a method by which biological imaging can raise the contrast in any imaging scheme.

Our method has many advantages. First, even after filtering, the photon budget per pixel is large (because of binning spectral-lifetime data). Second, the lack of computation makes the processing faster and a real-time-imaging option. Third, unknown samples can be probed with a quick anisotropy optimization and spectral collection to generate a contrast axis without any information of the sample. Similarly, known samples can be calibrated for the best polarization-spectral range for HDIM contrast. Fourth, the imaging implementation requires a FLIM system and a fiber as opposed to a costly spectral-setup. Our calibration setup uses two PMTs but can be achieved using a single PMT and a rotating emission polarizer. However, there are many challenges involved with the current implementation, and more research and optimization are required. This list includes the lack of a real-time FLIM analysis tool that can integrate the custom phasor or λ-τ based contrast. More research is necessary to integrate this marker into imaging as a contrast metric. Similarly, adding a mechanism to void parameter influence would also be helpful. This parameter cancelation can be achieved using (1) setting a circular polarization for removing anisotropy, (2) replacing the fiber with the free-space collection for removing spectral effects, and (3) summing the time-resolved axis to remove intensity decay information. This switching can be fully automated and can be useful for parameter separation. Another avenue of improvement is the lack of spectral variation in the fiber-based dispersion. Observing only the spectral peak but not the width and number of modes severely limits the spectral information encoded in our imaging scheme. More careful processing of spectral-lifetime analysis is necessary to achieve the exact width of the emission spectra. In this manuscript, we present single-cell analysis from one sample, but we have observed an increased contrast for every leaf sample we tested (n > 6). Additional ongoing experiments to show multiple extrinsic fluorescence markers and biochemical fingerprinting of those markers in a single pixel level would strengthen our method. Future work in these directions to optimize HD-Contrast Imaging are underway, and we hope this can become an unsupervised biomedical tool for fluorescence contrast.

We presented the fiber-based HDIM method that extracts an enhanced fluorescence contrast than lifetime, spectrum, or polarization could generate alone. However, there are challenges in this scheme of separation. Unlike full-resolution HDIM, when we selectively filter and encode information, there can be losses. For example, if the spectral shift contrast increases and lifetime-contrast decreases, they could cancel each other. A more detailed study into the sources of autofluorescence is necessary to address such anomalies. However, in our measurements with anthocyanins, we do not observe this. Future studies can identify and isolate the contrast source using a multiparametric collection like Raman, mass spectrometry, and others. As far as image contrast is considered, this method of encoding information provides improved contrast in biological imaging without losing photons.

## 5. Conclusions

This work presents a powerful hardware-based enhancement of fluorescence contrast that can easily be applied to any FLIM system providing hyperdimensional contrast in the imaging. This approach improves existing FLIM systems two-fold. (1) Any known contrast can be enhanced by tuning the anisotropy or spectral contrast parameters using the a priori knowledge of the pixels or molecules. (2) Unknown samples can be painted with an HDIM contrast score (without any a priori information) using phasor transforms or lambda-tau maps to reveal heterogeneity and structural details. The main advantages include (1) fiber-HDIM is a simple implementation, (2) fiber HDIM costs a small fraction of a typical multiparametric imaging setup, and (3) fiber-HDIM works faster and achieves real-time pixel-painting of hyperdimensional differences because it uses selective photon filtering and circumvents the computational load that needs to look a larger dataset. This scheme of imaging will help researchers better understand molecular heterogeneity than using conventional-FLIM alone.

## Data Availability

All data available upon request.
